# Risk factors for human infection with West Nile Virus in Connecticut: a multi-year analysis

**DOI:** 10.1186/1476-072X-8-67

**Published:** 2009-11-27

**Authors:** Ann Liu, Vivian Lee, Deron Galusha, Martin D Slade, Maria Diuk-Wasser, Theodore Andreadis, Matthew Scotch, Peter M Rabinowitz

**Affiliations:** 1 Yale Occupational and Environmental Medicine Program, Yale University School of Medicine, New Haven, CT, USA; 2 Yale School of Public Health, New Haven, CT, USA; 3 Connecticut Agricultural Experiment Station, New Haven, CT, USA; 4 Yale Center for Medical Informatics, Yale University School of Medicine, New Haven, CT, USA

## Abstract

**Background:**

The optimal method for early prediction of human West Nile virus (WNV) infection risk remains controversial. We analyzed the predictive utility of risk factor data for human WNV over a six-year period in Connecticut.

**Results and Discussion:**

Using only environmental variables or animal sentinel data was less predictive than a model that considered all variables. In the final parsimonious model, population density, growing degree-days, temperature, WNV positive mosquitoes, dead birds and WNV positive birds were significant predictors of human infection risk, with an ROC value of 0.75.

**Conclusion:**

A real-time model using climate, land use, and animal surveillance data to predict WNV risk appears feasible. The dynamic patterns of WNV infection suggest a need to periodically refine such prediction systems.

**Methods:**

Using multiple logistic regression, the 30-day risk of human WNV infection by town was modeled using environmental variables as well as mosquito and wild bird surveillance.

## Background

Human infection with West Nile virus (WNV) has emerged as a major public health problem in the US since its original detection in 1999. As cases of infection in both animals and humans have spread across North America, public health strategies have been developed to identify areas of increased risk of vector borne transmission of infection to humans [1-3]. One of the goals of such strategies has been to predict areas of increased human risk in order to take preventive actions such as mosquito control and public health messaging about protective behaviors. To varying degrees, predictive systems have relied on environmental and animal sentinel indicators to identify hotspots for human infection risk. These indicators can be divided into three main groups. Variables in the first group include land use and population density and are relatively static over a medium to long time interval, providing clues regarding likely mosquito habitat or opportunities for enzootic or zoonotic transmission [4,5]. The second type is more dynamic environmental variables including daily climate records of precipitation and temperature that vary over short time periods [6,7]. The third type is animal sentinel data including surveillance reports of vector mosquito infection and abundance and reports of infection in birds and other non-human vertebrates that could indicate human risk. While dead bird and trapped mosquito data are commonly used, data on other mammals such as horses have been used in some settings [8,9].

Despite the widespread occurrence of WNV infection in humans in certain years, there remains no agreed upon predictive model for WNV infection risk, and the relative predictive value of the different types of indicators categorized above remains unclear. We performed a multi-year analysis of risk factors for WNV infection in humans in the State of Connecticut for the years 2000-2005. Our aims were to determine the best predictive model for human WNV infection over this time period, to determine the relative value of the different classes of risk indicators, and to explore whether the patterns of risk factors were changing over this time interval.

## Results

**Figure 1:**
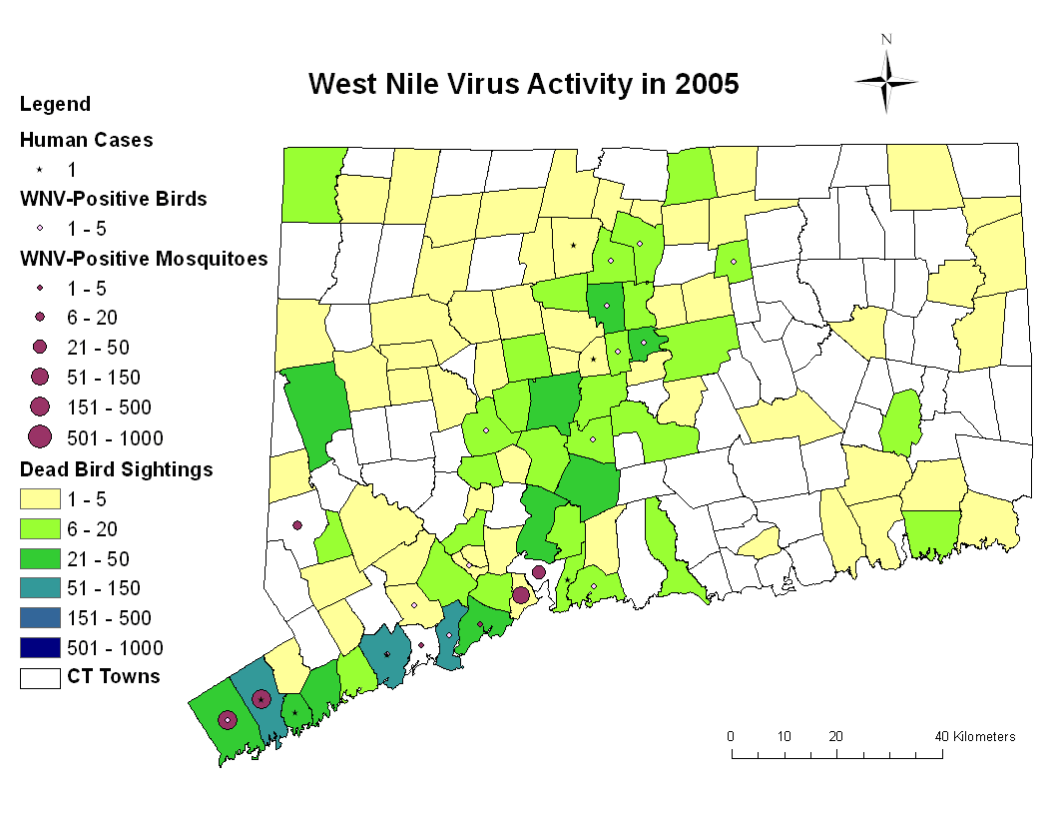
**Connecticut West Nile Virus surveillance results from 2005**. From [33].

The summary statistics for the static and dynamic variables examined are shown in Tables 1 and 2 as well as a risk map of some of these variables (Figure 1). For the land use variables, forested land cover displayed the highest mean percentage by town, by as much as an order of magnitude greater than many of the other land use variables. For the dynamic environmental variables, Growing Degree Days (GDD) showed an increasing pattern over the six-year period. For animal sentinel variables (bird and mosquitoes), there was significant temporal variation in the number of dead birds being reported annually to the surveillance system, with a peak of 3808 in 2003 declining to a low of 749 in 2005. The annual number of human WNV cases reported for the State also shows significant variability year to year, ranging from a low of 0 cases in 2004 to a peak of 17 cases in 2002.

**Table 1:** Static variables used to model human WNV infection risk in Connecticut from 2000-2005.

Variable		Mean	Standard Deviation (sd)
Land Use (%)	Commercial/Industrial	5.5	7.2
	Deep water	2.6	2.2
	Agricultural/soil/grass	19.5	9.9
	Forest	59.3	21.5
	Residential/commercial	8.3	9.7
	Rural/residential	1.6	0.9
	Wetlands	2.3	2.4
			
Population Density (persons/sq mile)		341.0	472.7

**Table 2:** Dynamic variables used to model human WNV infection risk in Connecticut from 2000-2005.

Statewide Climate Data	2000		2001		2002		2003		2004		2005	
	Mean	SD	Mean	SD	Mean	SD	Mean	SD	Mean	SD	Mean	SD
Daily Temperature	60.4	8.3	62.6	8.5	62.4	10.6	61.7	9.6	62.2	8.6	63.2	10.2
Yearly precipitation	14.3	7.6	11.0	8.2	14.8	6.1	17.6	6.8	14.5	6.7	15.9	13.8
Growing Degree Days	877.8	489.5	1057.4	588.1	1111.9	636.0	928.9	560.2	975.3	542.5	1055.8	655.4
												
**Animal Sentinel Data**	2000		2001		2002		2003		2004		2005	
	#		#		#		#		#		#	

Dead bird sightings	2448		3467		3766		3808		1573		749	
WNV positive birds	802		339		436		461		27		21	
WNV pos. mosquitoes	219		409		799		1060		924		518	
Mosquito abundance	32177		33184		35614		70262		44561		32064	
Human cases	1		6		17		15		0		6	

The results of the logistic regression modeling for the static and dynamic environmental variables of land use, climate, and population density, as predictors of human WNV infection risk are shown in Table 3. For the land use variables, only Agriculture/soil/grass (crop production areas, etc) remained significant in the model that adjusted for population density and climate. Population density remained significant as a predictor of human infection risk in the model, as did growing degree days and average temperature in the previous 30 days. Other studies [10,11] have found that urbanization is a risk factor for human WNV infection. Population density and residential/commercial land use are positively correlated and have both been linked to *Cx. pipiens *abundance in CT [12], precluding identification of the actual risk factor for human infection. To assess the potential bias in the selection of the lag period for average temperature, we re-ran the models using a range of lag periods. We found that using either 14, 60, or 90 day temperature averages did not improve the model's predictive power. The model that used only the environmental variables had a ROC/AUC value of 0.672 for the 2000-2005 period, indicating a moderate degree of predictive value.

**Table 3:** Model 1: Human WNV infection risk in Connecticut from 2000-2005 using environmental variables only.

	Unadjusted logistic regression				Adjusted logistic regression			
	OR	95% CI		p-value	OR	95% CI		p-value
**Land Use (%)**								
Commercial/industrial	1.08	1.06	1.10	<0.0001				
Deep water	1.02	0.91	1.14	0.7579				
Agriculture/soil/grass	1.04	1.01	1.07	0.0088	1.057	1.03	1.09	0.0003
Forest	0.95	0.93	0.96	<0.0001				
Residential/commercial	1.09	1.06	1.11	<0.0001				
Rural/residential	0.92	0.58	1.46	0.7325				
Wetlands	0.91	0.77	1.07	0.2615				
								
**Climate**								
Growing degree days	1.00	1.00	1.00	<0.0001	1.00	1.00	1.00	<0.0001
Avg. rainfall last 30 days	0.99	0.96	1.02	0.4247				
Avg. temp. last 30 days	1.18	1.11	1.26	<0.0001	1.20	1.13	1.27	<0.0001
								
**Other**								
Human population density	1.00	1.00	1.00	<0.0001	1.00	1.00	1.00	<0.0001
Year	1.02	0.91	1.14	0.7437				

The results of the predictive model that included only animal sentinel (mosquito and bird) data variables (model 2) is shown in Table 4. Here, the abundance of certain mosquito species, especially *Cx. pipiens*, was associated with an increased risk of human infection, while the abundance of *Cs. melanura *was associated with a decreased risk. When both mosquito abundance and bird cases were considered, only *Cx. pipiens *abundance remained a significant predictor of human risk. In addition, the presence of a WNV positive mosquito in a town during the previous 30 days was associated with increased human infection risk compared to an area where no trapping had been done. Similarly, the reporting of a dead bird in a town over the past 30 days, as well as WNV detected in a bird found in the town over the past 30 days were significant predictors of risk. The ROC value for the model that included the mosquito and bird sentinel data was 0.64.

**Table 4:** Model 2: Human WNV infection risk in Connecticut from 2000-2005 using animal-sentinel variables only.

	Unadjusted logistic regression				Adjusted logistic regression			
	OR	95% CI		p-value	OR	95% CI		p-value
**Mosquito abundance last 120 days (mosquitoes/trap day)**								
*Cs. melanura *> = 75% percentile	0.17	0.04	0.72	0.0156				
*Cs. melanura *not reported	0.08	0.04	0.16	<0.0001				
								
*Cx. pipiens *> = 75% percentile	6.25	3.14	12.45	<0.0001	3.00	1.47	6.13	0.003
*Cx, pipiens *not reported	0.22	0.09	0.54	0.0009				
								
*Cx. restuans *> = 75% percentile	2.55	1.34	4.87	0.0045				
*Cx. restuans *not reported	0.13	0.06	0.30	<0.0001				
								
*Cx. salinarius *> = 75% percentile	2.29	1.19	4.38	0.0127				
*Cx. salinarius *not reported	0.13	0.06	0.28	<0.0001				
								
*Ae. vexans *> = 75% percentile	4.43	2.30	8.55	<0.0001				
*Ae. vexans *not reported	0.18	0.07	0.41	<0.0001				
								
**Animal surveillance data in town last 30 days**								
All mosquitoes negative for WNV last 30 days (reference)	1.00				1.00			
Positive mosquitoes last 30 days	14.38	7.89	26.22	<0.0001	6.78	3.42	13.47	<0.0001
No mosquitoes recorded last 30 days	0.16	0.07	0.36	<0.0001				
Dead bird in past 30 days (reference: no birds reported)	7.43	2.88	19.15	<0.0001	2.80	1.19	6.56	0.02
WNV positive bird last 30 days (ref. no WNV positive birds)	13.18	7.75	22.41	<0.0001	3.94	1.96	7.90	0.0001

Table 5 shows the results of the model (model 3) that includes both environmental variables and animal sentinel data, and that analyzes these data over the entire time period of 2000-2005 as well as the three-year periods of 2000-2002 and 2003-2005. Over the entire time period, the predictive factors retaining significance include human population density, growing degree-days, average 30 day temperature, finding a WNV-positive mosquito in the town during the last 30 days, and the reporting of a dead bird or a WNV-positive bird in the previous 30 days. As in model 2, towns where no mosquito trapping had occurred over the past 30 days had a lower risk for human WNV cases. The ROC statistic for this overall model was 0.748, higher than both other models. The time stratification showed that the combined model performed better over the first three years of the study period (ROC/AUC = 0.87) than the second three year period (ROC/AUC = 0.521), and that mosquito data was more predictive in the first period while bird data, (both sightings of dead birds and WNV positive birds) was more significant in the predictive model during the second time period.

**Table 5:** Model 3: significant predictors of human WNV infection remaining in full model with both environmental and sentinel data- stratified by time period, by town.

	**2000-2002**				**2003-2005**				**2000-2005**			
	**OR**	**95% CI**		**p-value**	**OR**	**95% CI**		**p-value**	**OR**	**95% CI**		**p-value**
	
Agricultural Land Use	1.06	1.02	1.10	0.0026								
												
Population Density	1.00	1.00	1.00	<0.0001					1.00	1.00	1.00	<0.0001
Growing Degree Days (GDD)	1.00	1.00	1.01	0.0007	1.00	1.00	1.00	0.0010	1.00	1.00	1.00	<0.0001
												
Positive mosquito last 30 days (ref = mosquitoes neg last 30 days)	3.25	1.29	8.20	0.0124					3.08	1.67	5.68	0.0003
No mosquito testing last 30 days	0.35	0.07	1.68	0.1887					0.57	0.26	1.28	0.1761
												
Dead bird last 30 days					2.69	1.12	6.46	0.0266	2.48	1.28	4.78	0.0070
Positive bird last 30 days					3.11	1.28	7.56	0.0123	2.41	1.27	4.60	0.0074
												
Avg. temp last 30 days	1.49	1.29	1.71	<0.0001	1.09	1.00	1.18	0.0388	1.17	1.08	1.28	0.0003

## Discussion

Our longitudinal analysis of risk factors for human WNV infection found that a number of environmental variables including climate and population density, as well as the occurrence of WNV infection in mosquitoes and birds detected in active and passive surveillance efforts showed predictive value for human risk over a six-year time period.

Despite the attempt to assimilate data from a number of sources into a predictive model, this study had a number of limitations. A principal one was the small number of human cases in the state over the study period. In addition, the intensity of bird surveillance efforts appeared to have changed over the period, perhaps due in part to changing public perception about risk. Information about the implementation of mosquito control efforts was not incorporated into the current model, while the efficacy of control efforts for WNV is not well understood, such efforts could have affected the predictive ability of the surveillance data. The fact that towns with no mosquito trapping had lower risk of human cases could reflect the fact that trapping was performed in areas that were judged to be higher risk for WNV activity. However, such selective surveillance could result in detection bias. Despite these limitations, the final model was able to show significant relationships between risk factors and human risk. We believe that this was due in part to the data quality of the ongoing systematic mosquito surveillance program, as well as the quality of the wild bird surveillance program that continued over the entire study period.

For the static environmental variables, human population density remained a significant predictor even when adjusting for other environmental measures and sentinel data. This is in agreement with other studies that found positive association between human infection and urban/suburban environments versus more rural areas [10,11]. However, other studies such as Degroote et al [13] and Wimberly et al [14] suggest that the opposite is true in that less population density and rural areas is a risk factor for the disease. Perhaps geographical region had an impact on the discrepancy of these studies with some being in the Eastern part of the United States [10,11], and the others in the Western part [13,14]. For the land use/land cover variables, the data suggested a risk associated with the agricultural/soil/grass land use class during the period 2000-2002, however this variable did not remain significant across all the time periods. The grassland component of this class may represent more residential turf areas, which have been linked to *Cx. pipiens *[12]. In addition, agriculture has been shown to be a strong predictor of the abundance of *Ae. vexans *[12], a putative bridge vector of WNV from wildlife to humans.

We found significant associations between human infection risk and both growing degree-days and average temperature during the past 30 days. The importance of climate factors in our models is in agreement with a growing number of reports suggesting that real-time climate data can be useful in WNV risk prediction [9,15]. Temperature can affect mosquito emergence and developmental rates, the length for the pathogen extrinsic incubation cycle as well as human outdoor activity, all determinants of human infection risk.

While earlier studies of WNV infection in the western hemisphere have used dead bird surveillance to predict human infection risk [16], some researchers have found that mosquito surveillance may be more accurate than bird surveillance as a predictive tool [17]. Others have pointed out the potential for bias in relying on passive surveillance of dead birds, with human population density affecting the likelihood of reporting, and the fact that birds die from reasons other than WNV [18,19]. Our analysis of animal sentinel variables showed that both mosquito and bird surveillance data added significant amount of explanatory power to the environmental variables, and that dead birds remained significant in the final prediction model for the entire study period, even when adjusting for human population density. The relative value of mosquito and bird data appeared to vary by time period. Mosquito data were significant in the model for the period 2000-2002, while bird data were more significant in the model for the period 2003-2005. Some of this variation could be due to linkage between bird and mosquito virus prevalence Other changes could have included variation in level of concern among the public leading to decreased reporting of dead birds and impact of socioeconomic factors on reporting. These issues deserve further investigation in future studies of zoonotic disease surveillance systems.

When only animal sentinel data were considered, species-specific mosquito abundance showed a positive correlation for human infection risk, especially *Cx. pipiens*. Therefore the results of this study support the role of *Cx. pipiens *as enzootic and potentially a bridge vector in the Northeast United States.

We demonstrated that the predictive power of particular variables changed over the time period, suggesting that risk models should be continually updated. In addition, risk models must take into account the geospatial variation of West Nile Virus, since surveillance efforts have suggested that both the incidence of the disease, as well what factors are associated with increase WNV risk, can vary across different geographical locations in the United States.

A limitation to this study is the fact that some of the cases might not have been residents in Connecticut. Since this study focused on the summer months, there is a possibility that cases were vacationers from outside the study area. This could result in an overestimate of the risk of human WNV infection. Also, the state of Connecticut does not have universal healthcare. There is a possibility that un-reported cases occurred because some did not have access to medical care. If there were a large amount of WNV cases among the uninsured population in Connecticut this might have underestimated the risk of WNV infection.

Finally, other studies [7,20] discuss the importance of summer and winter months in relation to WNV risk. As future work, the authors want to explore the value of including winter months within the degree-day model as a possible improvement for examining WNV risk.

## Conclusion

Few published studies have looked at WNV risk factors over an extended period of time. Our longitudinal analysis of risk factors demonstrated that the relative value of specific risk factors might differ year to year as the pattern of infection evolves. We found decreases in the frequency of wild bird reports over the study period, suggesting changes in bird species abundance and/or changing host/reservoir dynamics of WNV infection in bird populations. These changes could also be due in part to changing surveillance practices and public awareness or concern over the time period. These dynamic patterns reinforce the need for prediction systems that are continually refined in order to adapt to changing environmental conditions and disease transmission patterns. Despite such fluctuations, however, and the sporadic and generally uncommon occurrence of human WNV cases in CT, the quality of the risk factor data allowed us to create a model with significant amount of explanatory power that could potentially help create a useful tool for public health monitoring and intervention for WNV risk.

Public health surveillance efforts are often limited by resource constraints, and there is therefore a need to evaluate the relative value of different strategies for prediction of a disease such as WNV. While integrated systems incorporating a variety of environmental and sentinel data streams may appear ideal, they are costly to maintain. In fact, the CT Dept of Public Health suspended their wild bird WNV surveillance in 2006.

The results of this study can contribute to the development of cost effective surveillance strategies for early detection and intervention for WNV risk in the future. Lessons learned with WNV surveillance can also shed light on the effective tracking of other zoonotic infections and vector borne infections.

## Methods

### Connecticut Department of Public Health (DPH) West Nile Virus Surveillance Data

The Connecticut WNV Surveillance Program has monitored WNV activity in humans, wild birds and mosquitoes since 2000. In Connecticut (and the United States as a whole), patients who seek medical care generally do so through visits to their primary care physician, specialist, or in more urgent cases, through emergency room visits. If the treating clinician (and/or laboratory) suspects or confirms a case of West Nile Virus, then they are expected to report the case to the Connecticut DPH. Between July and September, the period of greatest human WNV risk, the Connecticut DPH actively contacts hospitals across the state to more thoroughly identify patients with WNV, particularly concentrating on individuals diagnosed with encephalitis or meningitis. Between 2000 and 2005, 44 human cases of WNV in CT, by town, were reported to the Connecticut DPH.

Between 2000 and 2005, passive surveillance of bird mortality throughout the state was conducted with the cooperation of the public during the months of June through October. In the event of a dead bird sighting, residents were encouraged to call a specific telephone number designated by their local health departments. Data regarding the location and date of the sighting as well as the species of bird were submitted to the Connecticut DPH on a weekly basis and subsequently incorporated into an existing database. In addition to the records of sightings, dead birds submitted to local health departments from across the state were necropsied at the state veterinary diagnostic laboratory (on the campus of the University of Connecticut), and brain tissue samples were then tested for WNV using virus isolation. The majority of these birds were corvids (crows and jays). The policy of the surveillance program was that testing of dead birds from a given town ceased following confirmation of WNV in at least five separate birds; subsequently, further submissions of dead birds were discouraged and assigned lower priority for WNV testing. Therefore, we created a categorical variable for whether WNV positive birds had been identified in a town over the previous 30 days.

### Connecticut Agricultural Experiment Station (CAES) West Nile Virus Surveillance Data

Since 1999, CAES has conducted mosquito trapping, identification, and virus isolation at 91 statewide locations from June through October [21]. The trapping program was expanded from a pre-existing mosquito surveillance system established to monitor eastern equine encephalitis virus [22]. Approximately one-third of the sites were located in densely populated residential areas along the urban/suburban corridor that extends from Fairfield County along the coast to the Connecticut River and north into Hartford County. Traps in the five remaining counties were established in more rural settings. The number of traps per town varied from none to as many as five. Trapping was performed with dry ice baited CDC miniature light traps and gravid traps once every ten days at each trap location. Typically, traps were placed in the field during the late afternoon and retrieved the following morning [21]. Trapping locations were similar from year-to-year. Mosquitoes were identified according to species and processed for WNV infection in groups of up to 50 females and each pool was identified as positive or negative for West Nile virus using virus isolation.

Results from mosquito testing were stored in a database that included trap location, date of sampling, total number of mosquitoes tested, number of each species found, and whether or not the mosquito pool tested positive for WNV.

We created abundance variables in terms of mosquitoes per trap-day, calculated from the mosquito testing data, by adding together the total number of mosquitoes trapped within a town on the trap day and dividing by the number of traps within each town. In a similar fashion, we calculated, species-specific abundance data by town for the following species: *Culex pipiens, Culex salinarius, Culex restuans, Aedes vexans, and Culiseta melanura*, species believed to be some of the most important WNV vectors in CT [21]. Because trapping only took place in certain towns during the study period, we created a categorical variable to indicate towns where no testing took place.

### Connecticut DPH Population Density Data

The population density for each of the 169 towns was calculated for each year from 2000 to 2005. Estimated town populations from 2000-2005 were obtained from the Connecticut DPH based on the 2000 census and estimated growth rates. The denominator, land area for each town, was measured from a map of Connecticut town boundaries obtained from the University of Connecticut Magic Geospatial Data Resources [23].

### National Oceanic & Atmospheric Administration (NOAA) weather data

Daily surface observations of minimum (Tmin) and maximum (Tmax) temperature and precipitation data for 2000-2005 were obtained from the National Oceanic & Atmospheric Administration (NOAA) [24]. Landesman et al [25] have used precipitation data to model WNV risk. We linked each of the 169 towns in Connecticut to daily data from 16 Connecticut weather stations. All stations included precipitation data, but only 10 of the 16 weather stations recorded temperature data. Towns were assigned to the nearest weather station in terms of linear geographic distance. If towns were assigned to precipitation-only stations, they were additionally assigned to a station that recorded temperature data. For those days where there were missing observations, daily state averages were calculated from the remaining weather stations.

Trailing 30-day averages for temperature and precipitation, and daily cumulative growing degree-days (GDD) in excess of 10°C were calculated for each day within each town for analysis. GDD is a measure of seasonal accumulation of days where the temperature is over a certain threshold. GDD is a common metric used by agriculture industry to predict emergence of crops and agricultural pests during the growing season. In addition, studies such as Zou et al [26] have used it as a variable for WNV surveillance. Accumulation of GDDs begins with the first day in spring with a daily average temperature greater than 10°C. We used 10°C as the threshold since there is evidence that adult *Culex spp*. emergence is related to this threshold temperature [27]. GDD was computed by comparing the daily average temperature to a baseline of 10°C (See Equation 1)

#### Equation 1. Equation for calculating cumulative growing degree-days (GDD)

### University of Connecticut Magic Geospatial Data Land Use Land Cover Data

Using the 1995 land use/land cover classification map from the University of Connecticut Magic Geospatial Data Resources [28], the percent of different land use/land cover (LULC) classes were calculated for each of the 169 towns (In Connecticut, developed land increased by 1.2% between the time of this 1995 map and 2006 [29]. This change in urbanization during this period would likely have minimal effects on the end results.). The LULC classification map included a total of 28 categories. Using ArcGIS software version 9.1, the percentage of different land use/land cover (LULC) was determined by first collapsing the 28 total categories into 7 major categories: commercial/industrial, residential/commercial, rural/residential, agriculture/soil/grass, forest, deep water (e.g. some lakes), and wetlands. Commercial/industrial regions are used mainly to sell products and services. Examples include business districts or shopping centers [30]. Residential/commercial include high density and low density housing structures. Residential developments can also include residential strips adjacent to or extending from commercial urban centers [30]. Rural/residential encompass regions of scattered residential land use, such as farmsteads [30]. Wetlands consist of areas such as mudflats, swamps, and marsh [30]. Forest includes both deciduous, evergreen, and mixed forests [30].

### Elements of the Final Data Set

A master data set of human health data, avian surveillance data, mosquito surveillance data, climate factors and landscape variables was assembled and analyzed. For each day from January 1, 2000 through December 31, 2005, and for each of the 169 towns in Connecticut, the following information was included in the data set: daily occurrence of human cases of West Nile infection; occurrence and species of dead bird sighting; occurrence and species of WNV-positive bird; abundance of mosquitoes by species, and occurrence of WNV-positive mosquito pools. Also included in the data set were the population density; percentage commercial/industrial LULC; percentage of residential/commercial LULC; percentage of residential/rural LULC; percentage of agricultural/soil/grass LULC; percentage of forest LULC; percentage of deep water; percentage of total bodies of water; and percentage of wetlands for each town. Finally, the trailing 30-day average temperature and precipitation measurements, the cumulative GDD for each day as well as the trailing 120-day average of mosquito abundance during the specified time period were included. The choice of 30 day time lags for precipitation and temperature were somewhat arbitrary; time lags between 0 and 8 weeks have been used in other studies to predict West Nile vector abundance [31]. A 120-day trailing average for the mosquito data was selected to provide an estimate of the build-up of the mosquito population later in the season, which is when human cases are observed.

SAS software version 9.1 (SAS Institute, Cary, North Carolina) was used for all statistical analyses.

### Data Analysis

#### Logistic regression modeling

Three logistic regression models were created using different combinations of variables. The first model included only environmental variables (both static and dynamic). The second model used only animal sentinel (mosquito and bird) data. The third model combined both environmental and animal sentinel data. Unadjusted odds ratios of human infection risk were calculated using logistic regression for all independent variables described in the previous section.

Adjusted analysis was also conducted on the three models. To minimize collinearity, we included in the same model only those variables with a Pearson rank coefficient of less than 0.6. Highly correlated variables were considered as competing within the model. Forest and Agriculture/Soil/Grass were highly correlated. Population density was positively correlated with Residential/Commercial land use and very negatively correlated with Forest. Residential/Commercial land use and Commercial/Industrial land use were also highly correlated. Since both Population Density and Residential/Commercial land use were highly correlated with Forest, we used only one of these correlated land use variables (Forest) in the final logistic model. A backwards elimination procedure with a significance level to stay of p < = 0.05 was used to select significant variables remaining in the final models.

In order to explore temporal trends in the predictive value of different variables, we arbitrarily divided the study period into two three-year periods (2000-2002 and 2003-2005), and ran the models for each of those sub-periods. As a further check for temporal variation in the data, the multiple regression analyses described above were run using a generalized estimating equation (GEE). The GEE approach adjusts for the effect of temporal autocorrelation in time series data. The independent variables remaining significant in the models were identical for the logistic regression and the GEE models, with similar point estimates (data not shown).

To evaluate the explanatory power of each logistic regression model, we constructed ROC curves and calculated area-under-the-curve (AUC/c-statistic) estimates for each of the models using SAS [32].

## Competing interests

The authors declare that they have no competing interests.

## Authors' contributions

AL wrote the original manuscript, helped develop mathematical modeling and data analysis approaches, assembled data sets and the database. VL assembled climate data and performed additional analyses. DG performed statistical programming and analyses, and created tables. MDS provided expertise on database creation and statistical modeling. MDW assisted with revisions of manuscript and interpretation of mosquito data. TA collected and provided mosquito data. MS critically reviewed the manuscript and performed GIS analysis. PR conceived and oversaw database creation and analyses, and critically revised the analyses and the manuscript. All authors have read and approved the final manuscript.
